# The Practical Medicine Series of Year Books

**Published:** 1902-12

**Authors:** 


THE PRACTICAL MEDICINE SERIES OF YEAR BOOKS. Comprising ten volumes on the year's progress in medicine and surgery. Issued monthly under the general editorial charge of GUSTAVUS P. HEAD, M. D., Professor of Laryngology and Rhinology in the Chicago Post-Graduate Medical School. Volume IX. Physiology, Pathology, Bacteriology, Anatomy. Pathology edited by W. A. Evans, M. S., M. D., Professor of Pathology, College of Physicians and Surgeons, Chicago. Bacteriology edited by Adolph Gehrman, M. D., Professor of Bacteriology, College of Physicians and Surgeons, Chicago. Chicago: The Year Book Publishers, 1902. [Price, $1.25; entire series, $7.50.]

The present is one of a series of ten issued at monthly intervals and covering the entire field of medicine and surgery. This series is intended primarily for the general practitioner, the arrangement in several volumes being of special value to him.

This volume covers the departments of physiology, pathology, bacteriology, hygiene and anatomy From the study of the various antitoxins, their physiologic relationship and activity, the writer says: "In summarising the whole matter, a new biologic law has been established: foreign proteid molecules acting on certain living cells give rise to the production of chemical bodies having a specific relation to the substance under the influence of which they were produced; such bodies may be called antibodies." The other departments of this little work are admirably handled, short, concise abstracts being made of the leading scientific articles appearing in the medical literature.

The volume is an important one and shows what an unusual degree of activity is being expended in research work the world over.
W. C. K.

